# Philippines–Taiwan Oscillations and its connection to tropical cyclone frequency in the western North Pacific Ocean

**DOI:** 10.1038/s41598-018-35617-8

**Published:** 2018-11-29

**Authors:** Yu-Lin K. Chang, Yasumasa Miyazawa, Tsubasa Kodaira, Swadhin Behera

**Affiliations:** 1 Application Laboratory, Japan Agency for Marine-Earth Science and Technology, Yokohama, 236-0001 Japan; 2 Department of Ocean Technology, Policy, and Environment, Graduate School of Frontier Sciences, The University of Tokyo, Kashiwa, 277-8561 Japan

## Abstract

An atmospheric component of the Philippine–Taiwan Oscillations (PTOa) is used to examine its potential connection with tropical cyclone (TC) frequency in the western North Pacific in the period of 1979–2014. During positive PTOa years, more TCs are observed in the tropical western Pacific south of 18 °N where cyclonic wind anomaly appears. On the other hand, anti-cyclonic wind anomaly appears in the subtropics north of 18 °N and is associated with less TC formation there. The opposite wind vorticities in tropics/subtropics and associated variability in TC frequency reverse in negative PTOa phase. Besides, the negative contribution of ocean heat content suggests the relative importance of wind vorticity according to the oceanic cyclone genesis potential index. The PTOa provides a more direct explanation of the TC activity compared to other remotely linked phenomena at least for the past 36 years (1979–2014). Therefore, PTOa can potentially serve as a promising index for diagnosing or forecasting TC activity in the western North Pacific Ocean.

## Introduction

Tropical cyclones (TCs), one of the major natural disasters in the world, cause huge loss of lives and extensive damages to properties due to the strong winds, heavy rainfall, and storm surges^1^. Western North Pacific is the most active TC region in the world. There are about 26 TCs per year, accounting for more than 30% of global TCs^2^. The number of TCs formed in the region vary from year to year and that fluctuation is attributed to the atmosphere-ocean variation, often linked to some of the basin-scale phenomena in the Pacific Ocean. TC activity over the western North Pacific and its potential connection to El Niño and Southern Oscillation (ENSO^3^) had been addressed extensively in the previous studies^4–8^. It was suggested that TCs were more active during the canonical El Niño years as compared to the La Niña years^6–8^. TC frequency was also found to be significantly correlated with El Niño Modoki/La Niña Modoki (ENSO Modoki)^4,5,9^. The winter–spring Niño3.4 index had been proposed to predict the coming summer and autumn TC formation^8^. The Niño 3.4 index had also been found to be related to Australian TC genesis in south Western Pacific^10^.

Previous studies had hypothesized that wind may play a role in connecting the remote sea surface temperature (SST) in central or eastern Pacific to the TC activity in the western North Pacific Ocean^4,11^. Matsuura *et al*.^11^ proposed that heating of central Pacific SST associated with the western North Pacific cyclonic circulation occurred during high-frequency TC period. Chen and Tam^4^ found that heating of eastern Pacific SST was positively (negatively) correlated to TC frequency south (north) of 20 °N due to the existence of low-level dipole in wind anomaly over the western North Pacific. The previous study also suggested that El Niño is connected to the change of shear vorticity, western North Pacific subtropical high and monsoon trough, which further influences TC formation^8^. Although such relationships are viable, it is difficult to comprehend their direct impact on TC formations in the western North Pacific Ocean in the absence of a local mechanism. The local process may provide a more direct explanation of the TC activity compared to the remote teleconnections. Here we demonstrated such a possibility.

Among the local processes, the low-level wind vorticity is considered as one of the important factors influencing the TC formation^12^. Recent studies suggested that low-level vorticity is strongly related to TC genesis^13,14^. The newly proposed TC genesis potential index (GPI), using oceanic parameters, also uses the low-level wind vorticity for defining the index^15^. Therefore, we tried to find a possible local wind vorticity that could explain the TC activity in the western North Pacific. The Philippines–Taiwan Oscillations (PTO^16^, reference hereafter CO2012), a climate index defined based on the tropical and subtropical wind stress curl over western North Pacific is noted. PTO was developed to explain the ocean circulation^16,17^, and it had also been used in the fishery application studies in western North Pacific Ocean^18,19^. Owing to the PTO definition based on the wind stress curl, which is highly correlated to the low-level wind vorticity, the low-level wind vorticity based PTO (PTOa, the definition is given in next section) may potentially serve as a direct local index in explaining the western Pacific TC activity. The present work aims to investigate this connection between PTOa and TC frequency based on the atmospheric and oceanic reanalysis data. We also discuss a potential triggering mechanism of PTOa and its subsequent influence on the TC activity.

## Data and Methods

Tropical cyclone information from 1945 to 2014 is obtained from the best track data of the Joint Typhoon Warning Center (JTWC, http://www.usno.navy.mil/NOOC/nmfc-ph/RSS/jtwc/best_tracks/). Ocean heat content is derived from EMCWF ORAS4 ocean reanalysis at 1-degree horizontal resolution and covers the period from 1958 to present (https://www.ecmwf.int/en/research/climate-reanalysis/ocean-reanalysis). Wind data is based on National Centers for Environmental Prediction (NCEP) reanalysis II, with 2.5-degree resolution and extends the time period from 1979 to present (https://www.esrl.noaa.gov/psd/data/gridded/data.ncep.reanalysis2.html). The study period considered for the present work is from 1979 to 2014 that is when all data were available for the analysis.

PTO index follows the similar definition of CO2012, and is calculated as:

$$PTOa=\nabla \times {u|}_{zoneA}-\,\nabla \times {u|}_{zoneB}$$

In the present study, we replace the wind stress curl in CO2012 by the wind vorticity at 1000 mb (∇ × *u*), which is a more direct indicator for TC formation. Other definitions, like taking the wind vorticity difference between tropical (zoneA, 8 °N–13 °N, 130 °E–150 °E) and subtropical (zoneB, 22 °N–27 °N, 155 °E–180 °E) domains of western North Pacific, remain the same as CO2012. The updated atmospheric PTO using wind vorticity is called PTOa to distinguish it from the original PTO. A high correlation of 0.94 (p < 0.01) is noted between PTO and PTOa based on an expected close relationship between low-level wind vorticity and wind stress curl. PTOa index is derived from NCEP reanalysis II.

All data are organized into the monthly format. Monthly climatologies are calculated over the study period. To study different climate phases on interannual time scale, anomalies are computed by subtracting the monthly climatology from the monthly totals. Weighted composites were then made for the selected variables based on the time series of PTOa, following the formula given in CO2012

$$Composit{e}^{+}={\sum }^{+}(variable(t)\times PTOa(t))/{\sum }^{+}PTOa(t)$$

where *t* is time, ∑^+^ denotes the sum for positive phase (when PTOa was positive) only. A similar formula was used for the composites of negative phase (when PTOa was negative). The criteria for positive and negative phases refers to zero PTOa. The above formula reduces the influence of weak PTOa events and enhances the influence of major PTOa events. The TC formation location is defined where wind speed exceeds 39 mph (tropical storm intensity). The difference in TC frequency is accessed using the chi-squared test. The composite significance is examined by the student t-test. Both wind vectors and vorticity are considered while computing the significance of the wind field.

## Results

**Figure 1:**
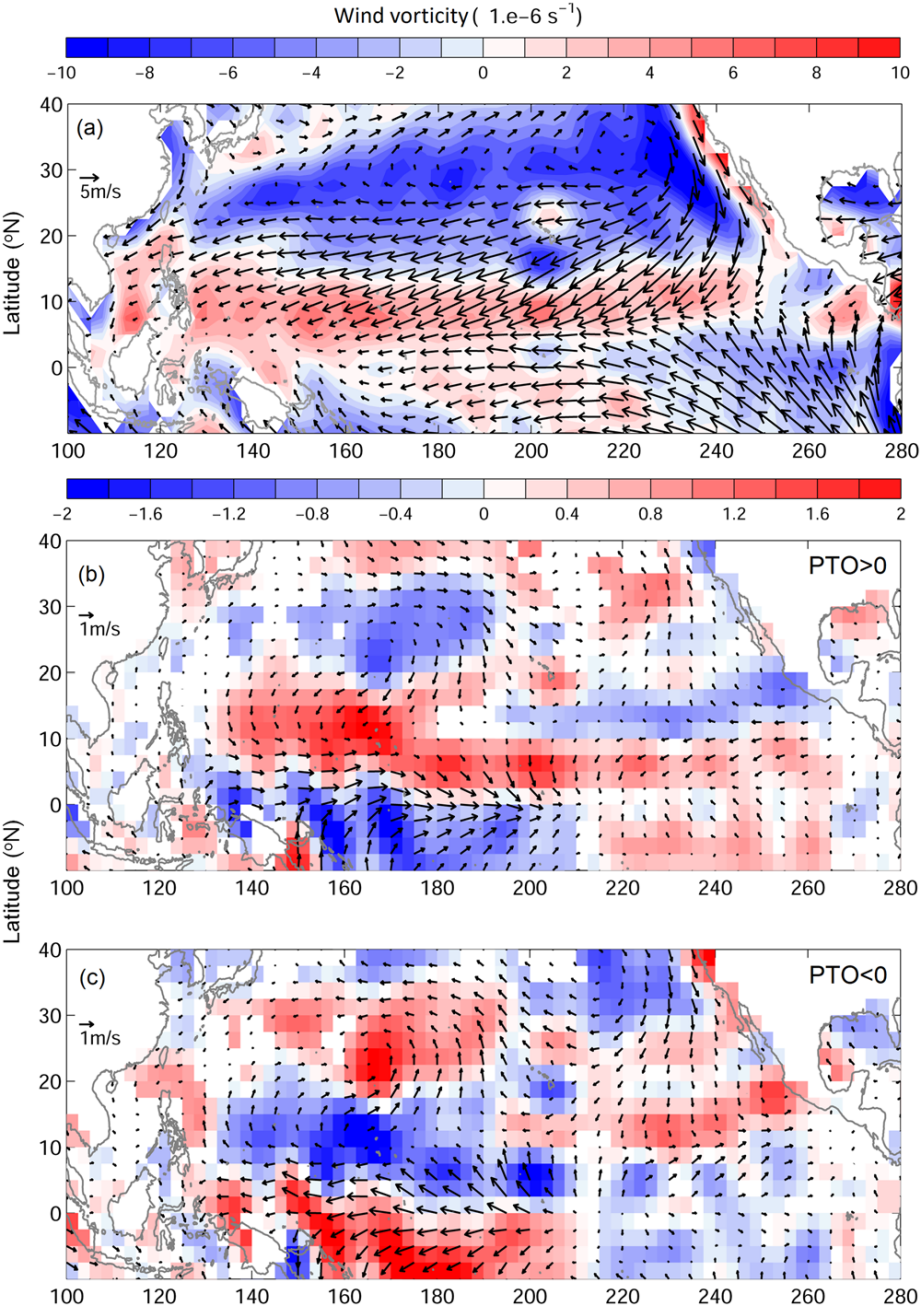
Wind (vector, m/s) and wind vorticity (color, 1/s) at 1000 mb for (**a**) annual mean from 1979 to 2014, and composite anomaly during (**b**) positive PTOa years, and (**c**) negative PTOa years. The blank area represents insignificant value.

The annual averaged low-level wind at 1000 mb in the tropical and subtropical western North Pacific are generally easterly (Fig. 1a). The corresponding wind vorticity changes sign around 16–18 °N, with positive (cyclonic) and negative (anti-cyclonic) vorticity to south and north of that band in the tropical and subtropical regions, respectively. During the positive PTOa years, the low-level cyclonic wind anomaly appears in the tropical western North Pacific with associated anti-cyclonic wind anomaly to its north (Fig. 1b). The positive vorticity anomaly in the tropics, extending from 5 °N to 18 °N, and from 140 °E to dateline may serve as a favorable condition for TC formation as per the criteria of GPI^12,15^. On the contrary, the negative vorticity anomaly north of 18 °N associated with the positive PTOa may potentially reduce the chances of TC formation. Vorticity anomalies are reversed between the tropics and the subtropics in the negative PTOa years (Fig. 1c). The anti-cyclonic wind anomaly occurs in the tropics and the cyclonic wind anomaly is observed in the subtropics. The effect of PTOa on TC formation during negative PTOa will also reverse, leading to less number of TCs in the tropical western Pacific, and more number of TCs in the subtropical region.

**Figure 2:**
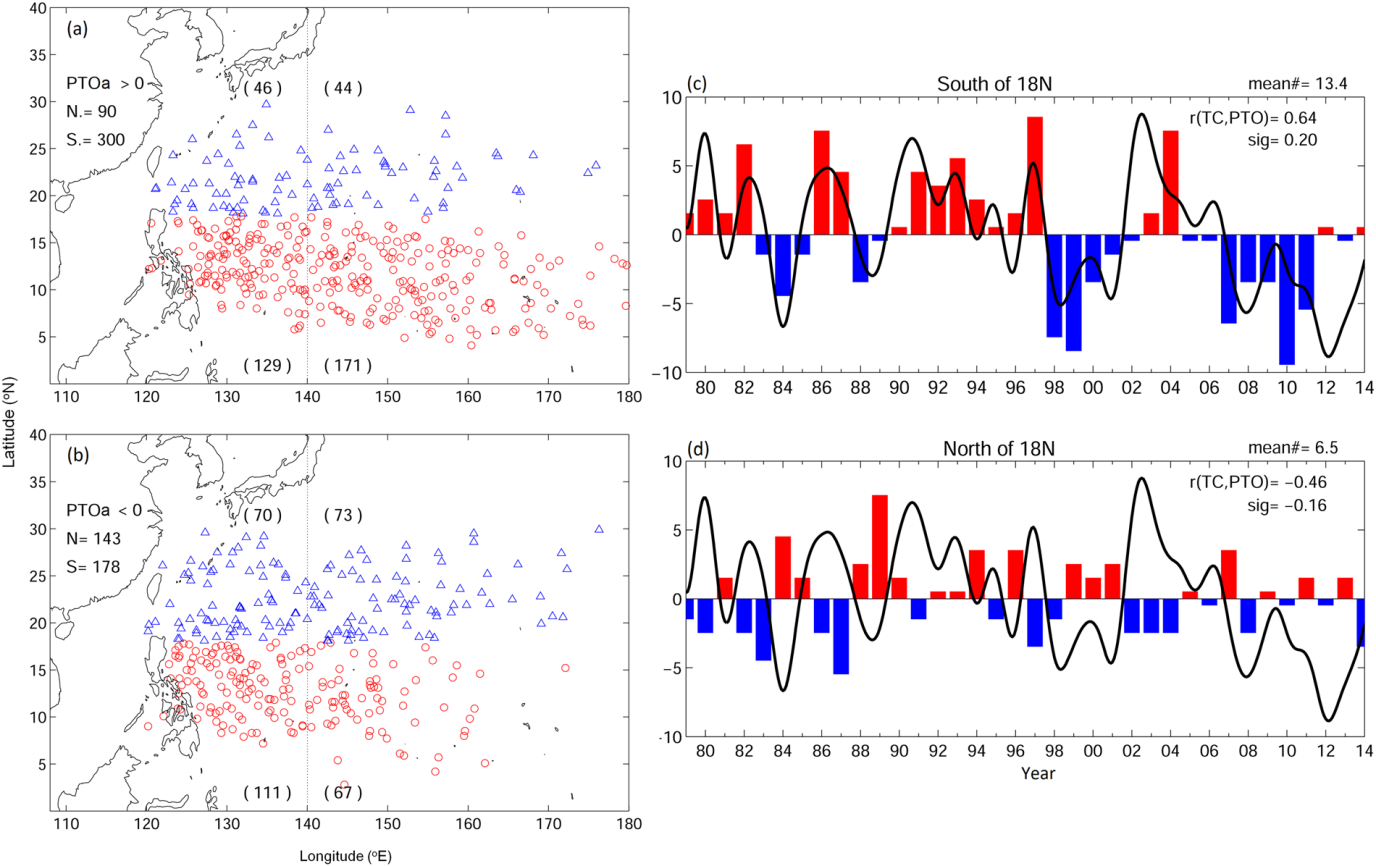
Tropical cyclone forming locations during (**a**) positive PTOa years, and (**b**) negative PTOa years. PTOa and tropical cyclone frequency from 1979 to 2014 in (**c**) south of 18N, and (**d**) north of 18N. Red and blue markers used in (**a**,**b**) represent south and north of 18N, respectively.

The plots of TC formation and distribution reveal the above inferences (Fig. 2a,b). More TCs form south of 18 °N during positive PTOa years than the negative PTOa years; 300 and 178 TCs were observed in positive and negative PTOa years, respectively. North of 18 °N shows opposite situation, fewer TCs are observed north of 18 °N in positive PTOa years than that in negative PTOa years; 90 TCs were formed in the positive phase and 143 TCs in the negative phase. The difference between the positive and negative PTOa years in tropical and subtropical regions are significant (p < 0.01). The seesaw in TC frequencies during the two PTOa phases corresponds to the change in wind vorticity (Fig. 1b,c). More TCs occur in positive wind vorticity anomaly region as compared to that in negative wind vorticity anomaly region associated with both phases of PTOa.

The TC activities are also seen to spatial vary within the domain of the PTOa. In the tropical region south of 18 °N, the TC frequency differences between the two PTOa phases mainly appear to the east of 140 °E. TC frequency in positive PTOa years is more than double of that in negative PTOa years (171 versus 67). That variation corresponds to the favorable cyclonic wind anomaly during positive PTOa years and the unfavorable anti-cyclonic wind anomaly in negative PTOa years (Figs 1 and 2). The wind vorticity is reversed west of 130 °E, TC frequency difference between positive and negative PTOa years becomes smaller but remains significant (p < 0.01, 129 versus 111). In the subtropical western Pacific north of 18 °N, wind vorticity keeps the same sign from 120 °E to the dateline. The anti-cyclonic wind anomaly during positive PTOa years corresponds to the less TC frequency in both west and east of 140 °E during positive PTOa years, and vice versa for negative PTOa years.

Apart from the significant differences in the composites of TC genesis regions, between the two phases of PTOa, the time series of TC frequency south (north) of 18 °N is found to be positively (negatively) correlated (significantly p < 0.05) with PTOa index (Fig. 2c,d). The correlation is 0.64 south of 18 °N and is −0.46 north of 18 °N. It is found that the correlation between TC frequency and PTOa is higher compared to that of the ENSO Modoki index (EMI) and canonical ENSO index (Niño 3 index). Chen and Tam^4^ reported a correlation coefficient of 0.31 between TC frequency and EMI. The corresponding correlation becomes −0.13 for Niño 3 index. Although improvements in the correlation coefficients were found after changing the domains of the TC genesis in their study, PTOa has a better correlation with TC frequency in western North Pacific. Moreover, since the PTOa index is based on the wind vorticity, PTOa provides a direct mechanism to explain the TC cyclogenesis. We also compared this relationship with that of GPI. The range of correlations among GPI^12^, oceanic GPI^15^ and observed TC frequency is between 0.39 and 0.54^15^. That range of correlation values are comparable to the correlations values of PTOa and TC discussed earlier. However, considering the complexity of deriving GPI with several ocean-atmosphere parameters, the PTOa would be a better choice to analyze and predict TCs in western North Pacific.

**Figure 3:**
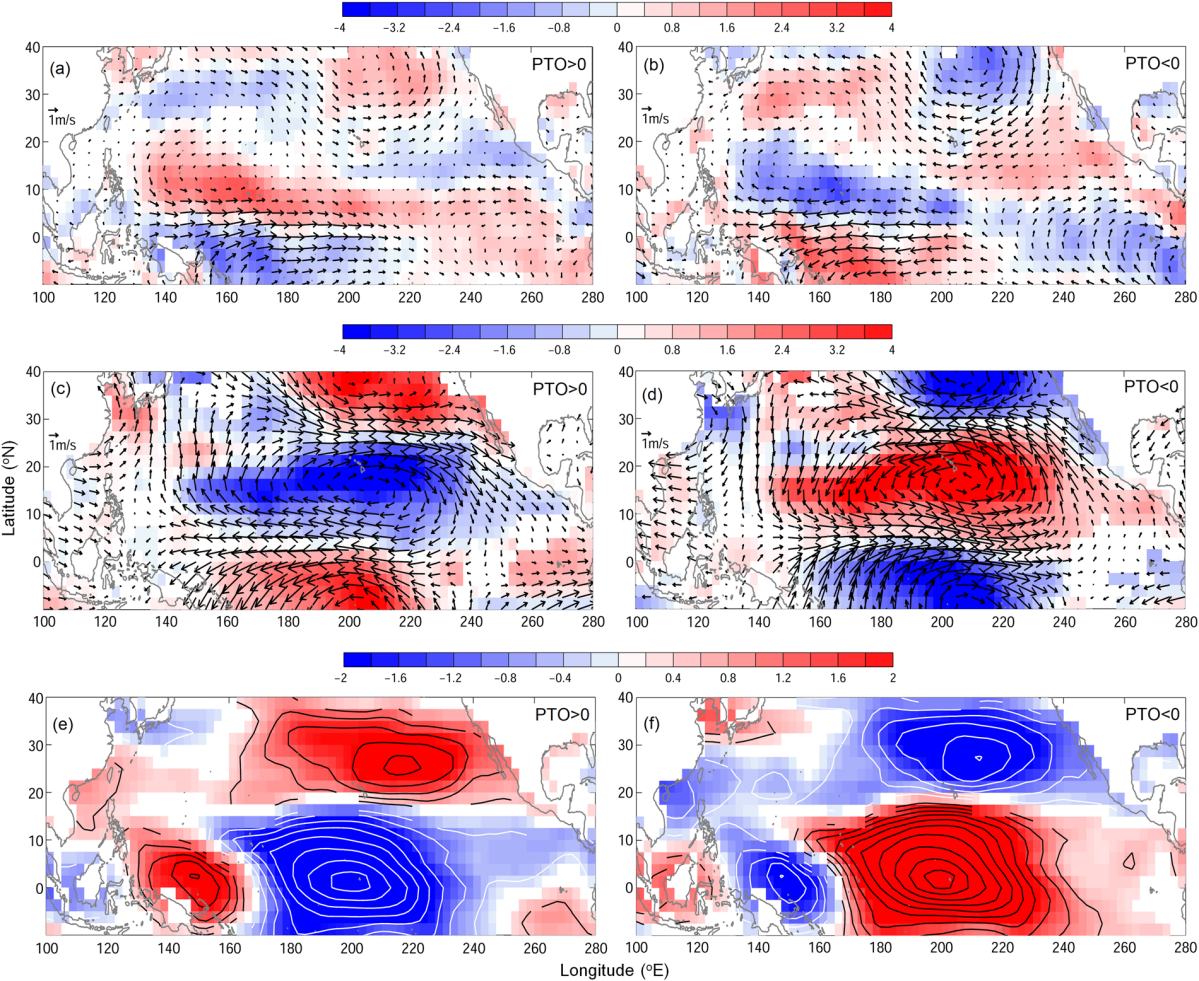
Composites of wind (vector, m/s) and wind vorticity (color, 1/s) anomalies at (**a**,**b**) 850 mb, (**c**,**d**) 200 mb, and (**e**,**f**) vertical wind shear anomaly (m/s) for (**a**,**c**,**e**) positive PTOa years, and (**b**,**d**,**f**) negative PTOa years. Insignificant values are not shaded (blank).

The development of wind vorticity anomaly corresponding to PTOa changes the low-level wind field in the western North Pacific. That change in the low-level wind filed modifies the vertical wind shear and influences the TC formation^12^. For example, during the positive PTOa years, the cyclonic wind anomaly in tropical region, associated with PTOa, weakens the low-level (850 mb) easterly wind near the equator (Fig. 3a). Furthermore, that tropical cyclonic anomaly together with the subtropical anti-cyclonic wind anomaly strengthens the easterly wind around 20 °N. Meanwhile, a triple vorticity pattern appears in the upper-level wind at 200 mb with two in lower latitudes extending to the western Pacific (Fig. 3c). Those upper-level vorticities weaken the upper-level westerly wind near the equator and strengthen the westerly wind between 20 °N and 30 °N. These wind changes in near surface and upper-level, therefore, give rise to a negative (positive) vertical wind shear south (north) of 18 °N during positive PTOa years. The negative vertical shear, a favorable condition for the TC formation, extends from 140 °E to dateline between 10 °N and 18 °N and enhances TC activity in that region. At this time, an unfavorable positive vertical wind shear develops north of 18 °N that reduces TC frequency there (Figs 2a and 3e). The condition is reversed during negative PTOa years (Fig. 3b,d and f). The vertical wind shear, that affects the TC activity, discussed here are seen to be associated with either phase of the PTOa, which is based on the low-level wind anomalies. It is possible that the upper-level winds are affected by the near surface conditions. However, the mechanism needs further investigation perhaps using sophisticated ocean-atmosphere coupled models.

**Figure 4:**
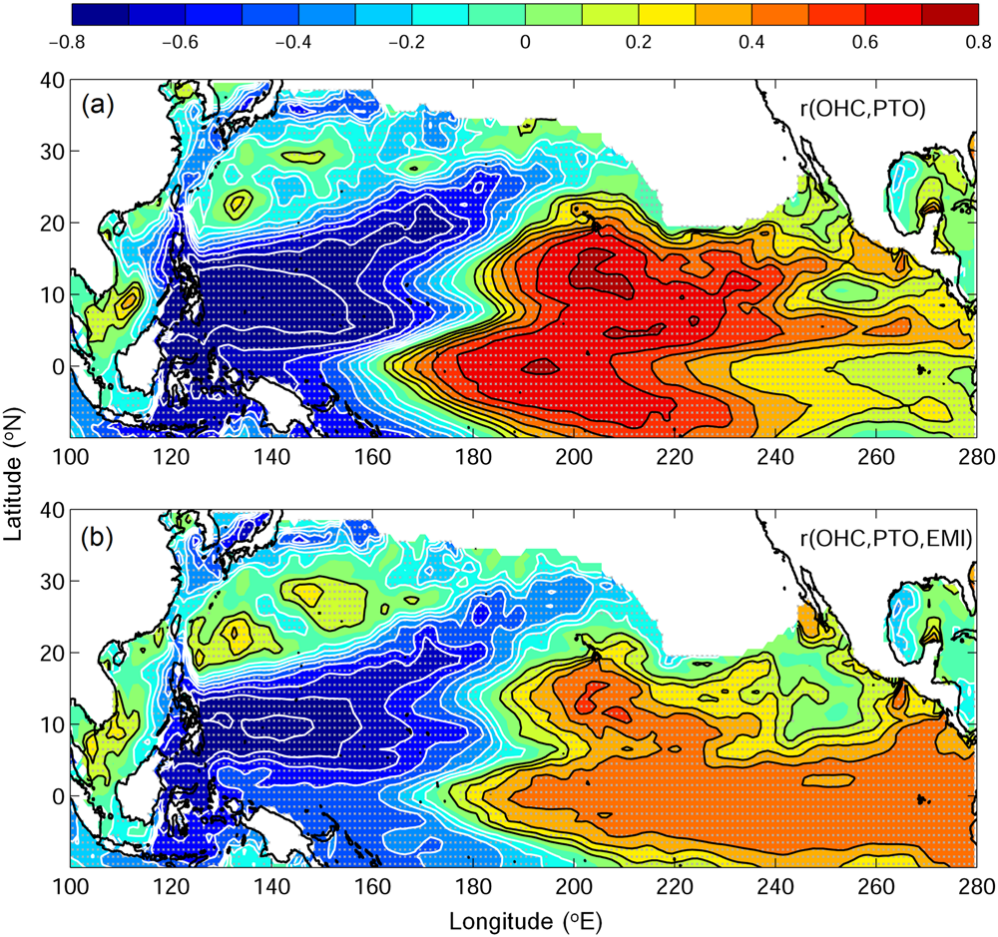
The correlation coefficient between (**a**) OHC and PTOa, and (**b**) partial correlation between OHC and PTOa by removing the effect of El Niño Modoki. Blank area in mid-latitude is caused by the inexistence of 26° isotherm. Gray dots are superimposed on the significant correlation locations.

The correlation between PTOa and ocean heat content (OHC)^20^ shows a local connection over the western North Pacific Ocean (Fig. 4a,b). This covariation in OHC in western Pacific could be explained by the wind vorticity of PTOa through the Ekman convergence and divergence mechanism^16^. During positive PTOa years, the cyclonic wind vorticity in tropics and the anticyclonic wind vorticity in subtropics result in the upward and downward isopycnals in corresponding regions and therefore the lower and higher OHC, respectively. The condition is reversed during negative PTOa years, leading to the higher OHC in tropics, and lower OHC in subtropics. A recent study by Zhang *et al*.^15^ proposed a new GPI based on absolute vorticity at 1000 mb, mean ocean mixed-layer temperature, longwave radiation, and depth of 26-degree isotherm. During positive PTOa years when more TC formation is observed in tropical western Pacific, the corresponding OHC anomaly is negative and the wind vorticity anomaly is positive (Fig. 1b and 4a). Negative OHC infers the last three terms of GPI which are negative and therefore the positive wind vorticity dominates, in order to favor more TC formation. The same analogy applies to negative PTOa years in an opposite sense. The relative importance of wind vorticity to the TC formation also suggests PTOa could be a promising index for diagnosing or forecasting TC frequency in the western North Pacific.

## Summary and Discussion

The present study explores the potential of using an updated local western North Pacific climate index, the atmospheric Philippines–Taiwan Oscillations (PTOa), in explaining tropical cyclone (TC) activity in the western North Pacific based on the past 36 years (1979–2014) atmospheric and oceanic reanalysis data and observations. The dipole pattern in the wind vorticity of PTOa serves respectively as a favorable and unfavorable condition for TC formation in tropical and subtropical western North Pacific. TC activities correspond well to the dipolar wind vorticity structure associated with PTOa. During positive PTOa period when cyclonic and anti-cyclonic wind anomaly occurs, respectively, at the tropical and the subtropical western North Pacific, more TC is observed in tropics south of 18 °N, and less TC is found in subtropics north of 18 °N. The condition is reversed during negative PTOa years. Besides, the negative contribution of ocean heat content (OHC) used in a recently developed genesis potential index (GPI)^15^, to TC activity, our study suggests the relative importance of wind vorticity in applying PTOa to understand TC activity. Compared to the remote connections arising from basin-scale phenomena, such as canonical ENSO or ENSO Modoki in eastern or central Pacific, the tropical and subtropical wind vorticity of PTOa provides a more direct mechanism to explain the TC activity locally. Moreover, PTOa shows a higher correlation to TC frequency compared to ENSO indices over the past 36 years (1979–2014). Besides, the oscillation of TC formation between the tropics and subtropics is associated with dipoles in wind vorticity, and that may explain the relatively low correlation between TC frequency and canonical ENSO/ENSO Modoki^4^ indices. This is also apparent in Fig. 2c,d; the phase of positive and negative TC frequency anomalies closely follows the index of PTOa irrespective of ENSO variability. Moreover, when the entire western Pacific combining the TC genesis anomaly in tropics and subtropics, as usually considered in previous studies, is taken in to account the signals from tropics and subtropics get canceled out leading to the misrepresentation of the signal and weak relationship with climate indices.

**Figure 5:**
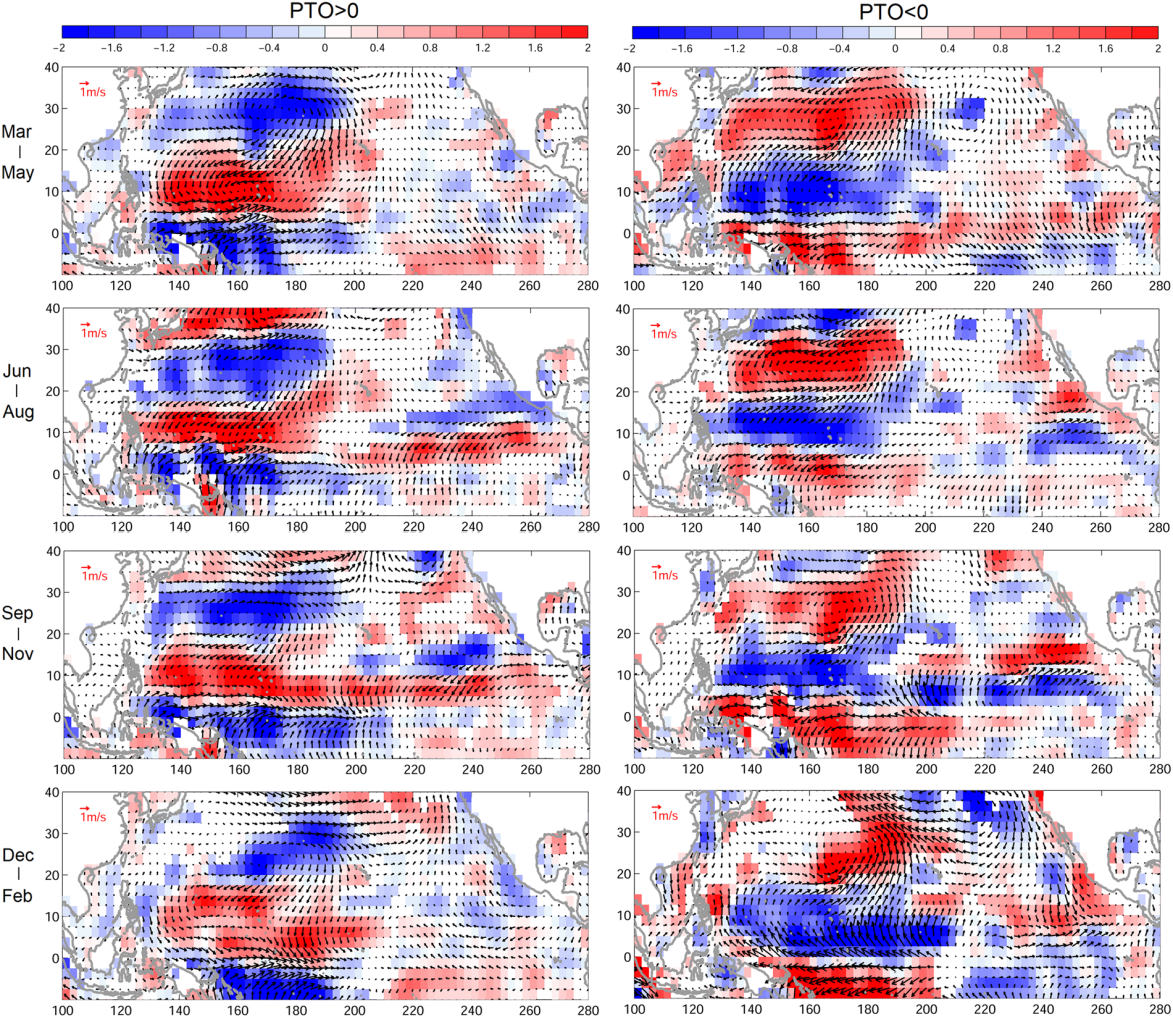
Seasonal composites of wind (vector, m/s) and wind vorticity (color, 1/s) anomalies at 1000 mb for (top to bottom) spring, summer, autumn, and winter during (left) positive PTOa years, and (right) negative PTOa years. Insignificant values are not shaded (blank).

Previous studies suggested that strong El Niño event could drive eastward extension of monsoon trough leading to anomalous wind vorticity and its effect on TC genesis^21,22^. The eastward extended TC distribution and the corresponding cyclonic vorticity can also be seen south of 18 °N during positive PTOa years (Figs 1b and 2a), in which the summer and early autumn cyclonic vorticity could be associated with the monsoon trough movement. Although TCs mainly form in summer and autumn, that corresponds to the existence of monsoon trough, TCs were indeed observed during all seasons. The wind vorticity dipole of PTOa is a persistent feature (Fig. 5), the subtropical and tropical wind vorticity anomaly can be seen nearly year-round, which can potentially be a predominant index for TC formation and could certainly capture its variations in summer and autumn seasons.

The classic GPI index^12^ is derived based on four major terms, including absolute vorticity, relative humidity, vertical wind shear, and the potential intensity. The potential intensity needs to be further calculated from sea surface temperature, mean outflow temperature, convective available potential energy, boundary layer air, and other coefficients. Recent updated GPI^15^ introduced a simpler formula with three oceanic parameters and wind vorticity as listed in the previous session. Although GPI index considers more potential factors for TC genesis, not all the parameters are easy to obtain, and the actual correlation between GPI and observed TC frequency is not high due to nonlinearity or cross-interactions among different factors of GPI. PTOa is an easy access index, which only requires wind information in comparison to the GPI index, and it also performs an equal or higher correlation to TC frequency than GPI. PTOa can potentially be an adequate index for diagnosing or forecasting TC frequency in the western North Pacific.

Apart from TC formation, PTOa may also influence TC track in the western Pacific through the wind system changes. We notice that there is 35–60% more TC approach Japan and Korea during positive PTOa years. A previous study suggested that more TC is found southeast of Japan the year before El Niño event^7^, and PTO was noticed to lead ENSO Niño 3.4 index by 5 months^16^. How PTOa affect TC track requires investigation in future work.

The questions of how does PTO form or what triggers PTO remains unclear. PTO is noted to be related to Pacific climate indices^16^ (Fig. 1). A positive correlation between PTOa and OHC appears in central Pacific corresponding to the central pole of ENSO Modoki (Fig. 4). This was also observed in CO2012 who suggested that PTO has the highest correlation with ENSO Modoki among Pacific climate indices. Indeed, previous studies had shown the central Pacific warming associated with cyclonic wind circulation in western Pacific based on modelling or reanalysis data^4,5,23^. As El Niño Modoki leads PTO by 0–3 months^16^, El Niño Modoki could be one of the major triggering mechanism of PTO. The teleconnection mechanism could be a Matsuno-Gill response to precipitation anomalies^24,25^ associated with the El Niño Modoki. However, that mechanism alone could not explain all the PTO variation and the sustenance of the PTO in western North Pacific as noticed by CO2012. Other factors, such as ENSO, monsoon and local air-sea interactions could be important for its formation and sustenance. It should be noted here that the present study is based on the reanalysis data with obvious limitations as compared to the real observed data^26,27^. So, with that caveat, further investigations are needed to understand the detailed mechanism of PTO formation perhaps by using a regional or a global ocean-atmosphere coupled model.

## References

[1] Shultz JM, Russell J, Espinel Z (2005). Epidemiology of Tropical Cyclones: The Dynamics of Disaster, Disease, and Development. Epidemiologic Reviews.

[2] Landsea, C. W. *Climate variability of tropical cyclones: Past*, *Present and Future*. 220–241 (2000).

[3] Philander, S. G. *El Niño*, *La Niña*, *and the Southern Oscillation*. (Academic Press, 1989).

[4] Chen Guanghua, Tam Chi-Yung (2010). Different impacts of two kinds of Pacific Ocean warming on tropical cyclone frequency over the western North Pacific. Geophysical Research Letters.

[5] Pradhan, P. K., Preethi, B., Ashok, K., Krishnan, R. & Sahai, A. K. Modoki, Indian Ocean Dipole, and western North Pacific typhoons: Possible implications for extreme events. *Journal of Geophysical Research: Atmospheres* **116**, 10.1029/2011JD015666 (2011).

[6] Chan JCL, Liu KS (2004). Global Warming and Western North Pacific Typhoon Activity from an Observational Perspective. Journal of Climate.

[7] Chan, J. C. L. Tropical Cyclone Activity over the Western North Pacific Associated with El Niño and La Niña Events. *Journal of Climate* **13**, 2960–2972, doi:10.1175/1520-0442(2000)013<2960:tcaotw>2.0.co;2 (2000).

[8] Wang, B. & Chan, J. C. L. How Strong ENSO Events Affect Tropical Storm Activity over the Western North Pacific. *Journal of Climate* **15**, 1643–1658, doi:10.1175/1520-0442(2002)015<1643:hseeat>2.0.co;2 (2002).

[9] Ashok, K., Behera, S. K., Rao, S. A., Weng, H. & Yamagata, T. El Niño Modoki and its possible teleconnection. *Journal of Geophysical Research: Oceans* **112**, 10.1029/2006JC003798 (2007).

[10] Ramsay HA, Leslie LM, Lamb PJ, Richman MB, Leplastrier M (2008). Interannual Variability of Tropical Cyclones in the Australian Region: Role of Large-Scale Environment. Journal of Climate.

[11] Matsuura T, Yumoto M, Iizuka S (2003). A mechanism of interdecadal variability of tropical cyclone activity over the western North Pacific. Climate Dynamics.

[12] Emanuel, K. A. & Nolan, D. S. In *26th Conf*. *on Hurricanes and Tropical Meteorology* 240–241 (Amer. Meteor. Soc., Miami, FL., 2004).

[13] Aiyyer A, Molinari J (2008). MJO and Tropical Cyclogenesis in the Gulf of Mexico and Eastern Pacific: Case Study and Idealized Numerical Modeling. Journal of the Atmospheric Sciences.

[14] Wu L, Takahashi M (2018). Contributions of tropical waves to tropical cyclone genesis over the western North Pacific. Climate Dynamics.

[15] Zhang Min, Zhou Lei, Chen Dake, Wang Chunzai (2016). A genesis potential index for Western North Pacific tropical cyclones by using oceanic parameters. Journal of Geophysical Research: Oceans.

[16] Chang Y-L, Oey L-Y (2012). The Philippines–Taiwan Oscillation: Monsoonlike Interannual Oscillation of the Subtropical–Tropical Western North Pacific Wind System and Its Impact on the Ocean. Journal of Climate.

[17] Wang J, Oey L-Y (2014). Inter-annual and decadal fluctuations of the Kuroshio in East China Sea and connection with surface fluxes of momentum and heat. Geophysical Research Letters.

[18] Chang Y-L, Sheng J, Ohashi K, Béguer-Pon M, Miyazawa Y (2015). Impacts of Interannual Ocean Circulation Variability on Japanese Eel Larval Migration in the Western North Pacific Ocean. Plos One.

[19] Hsu AC, Xue H, Chai F, Xiu P, Han Y-S (2017). Variability of the Pacific North Equatorial Current and its implications on Japanese eel (Anguilla japonica) larval migration. Fisheries Oceanography.

[20] Leipper, D. F. & Volgenau, D. Hurricane Heat Potential of the Gulf of Mexico. *Journal of Physical Oceanography* **2**, 218–224, doi:10.1175/1520-0485(1972)002<0218:hhpotg>2.0.co;2 (1972).

[21] Wu L, Wen Z, Huang R, Wu R (2012). Possible Linkage between the Monsoon Trough Variability and the Tropical Cyclone Activity over the Western North Pacific. Monthly Weather Review.

[22] Wu L, Zhang H, Chen J-M, Feng T (2018). Impact of Two Types of El Niño on Tropical Cyclones over the Western North Pacific: Sensitivity to Location and Intensity of Pacific Warming. Journal of Climate.

[23] Ashok, K., Iizuka, S., Rao, S. A., Saji, N. H. & Lee, W. J. Processes and boreal summer impacts of the 2004 El Niño Modoki: An AGCM study. *Geophysical Research Letters* **36**, 10.1029/2008gl036313 (2009).

[24] Gill AE (1980). Some simple solutions for heat‐induced tropical circulation. Quarterly Journal of the Royal Meteorological Society.

[25] Matsuno T (1966). Quasi-Geostrophic Motions in the Equatorial Area. Journal of the Meteorological Society of Japan. Ser. II.

[26] Bosilovich, M. G., Kennedy, J., Dee, D., Allan, R. & O’Neill, A. In *Climate Science for Serving Society: Research*, *Modeling and Prediction Priorities* (eds Asrar, G. R. & Hurrell, J. W.) 51–71 (Springer Netherlands, 2013).

[27] Parker WS (2016). Reanalyses and Observations: What’s the Difference?. Bulletin of the American Meteorological Society.

